# Audit of Quality of General Practitioner (GP) Referrals to a Local Memory Service in South Sefton

**DOI:** 10.1192/bjo.2024.631

**Published:** 2024-08-01

**Authors:** Abosede Adegbohun, Shelton Sibanda, Salman Zafar, Sudip Sikdar

**Affiliations:** Merseycare NHS Foundation Trust, Liverpool, United Kingdom

## Abstract

**Aims:**

To assess the quality of General Practitioner (GP) referrals to a Local Memory Service in South Sefton – a reaudit.

**Methods:**

The quality of GP referrals received from primary care to the Memory Clinic at South Sefton Neighbourhood Centre (SSNC), Mersey Care NHS Foundation Trust, was assessed over three months. This reaudit was based on an initial similar audit conducted in 2019 of 106 GP referrals to SSNC.

The GP’s documented history and duration of memory loss, collateral history, and the impact of the patient's memory loss on activities of daily living (ADLs) were analysed. Also explored were the cognitive tests, physical examination, and completeness of blood investigations.

The expected standard for completeness was set at 100%. Achieved compliance for each parameter was graded 95% and above (green), 75% to 94% (yellow), and below 75% (red).

**Results:**

106 GP referrals were received in the SSNC Memory Service between June and August 2022. About 86% of the referrals had a history of memory loss noted by the referring GPs, while only 46% commented on the duration of memory loss. We observed increased documentation regarding the patient's history of memory loss, physical health status and cognitive testing. On the other hand, there was an 8% reduction in the referrals regarding the impact of memory loss on activities of daily living in comparison to the initial audit done in 2019.

About a quarter of all the GP referrals were accepted based on the information the GP provided on the first referral letter sent to the service. On the contrary, 70 referrals were either considered inappropriate or declined outright. Alternative diagnostic advice was given to the referring GPs in 12, and the GP asked to provide additional information in 9 of these 70 referrals. After the GP offered further details, 17 initially rejected referrals were accepted for assessment.

**Conclusion:**

Even though there were some observed improvements in the information GPs provided on referrals made to the local memory service in 2022 compared with 2019, this still fell drastically below the expected standard. The finding from this re-audit process brings to the fore the need for improved partnerships between memory services professionals and GP colleagues.

A new referral proforma has been designed in collaboration with the local Integrated Care Board (ICB), detailing essential information that needs to be documented by the GP before a referral is sent to memory services

